# Crossing the chasm from model performance to clinical impact: the need to improve implementation and evaluation of AI

**DOI:** 10.1038/s41746-022-00572-2

**Published:** 2022-03-03

**Authors:** Jayson S. Marwaha, Joseph C. Kvedar

**Affiliations:** 1 Beth Israel Deaconess Medical Center, Boston, MA USA; 2 Harvard Medical School, Boston, MA USA; 3 Mass General Brigham, Boston, MA USA

**Keywords:** Outcomes research, Health services

Artificial intelligence (AI) has been the subject of considerable interest for many years for its potential to improve clinical care—yet its actual impact on patient outcomes when deployed in clinical settings remains largely unknown. In a recent systematic review by Zhou et al.^1^, the authors surprisingly show that its impact so far has been quite limited. They reviewed 65 randomized controlled trials (RCTs) evaluating AI-based clinical interventions and found that there was no clinical benefit of using AI prediction tools compared to the standard of care in nearly 40% of studies. Among a subset of trials that the authors identified as having a low risk of bias, the clinical benefit of using deep learning (DL) predictive models over traditional statistical (TS) risk calculators was only minimal, and there was no benefit in using machine learning (ML) models over TS tools. Somewhat counterintuitively, most of the AI tools in these trials exhibited an excellent area under the receiver operating characteristic (AUROC; a common performance metric for predictive models) during development (median AUROC 0.81, IQR 0.75–0.90) and validation (median AUROC 0.83, IQR 0.79–0.97): a humbling reminder that robust predictive utility does not guarantee clinical impact at the bedside. As the science of building accurate predictive models progresses, our ability to translate these advancements into real-world clinical utility remains comparatively limited. How can we bridge this gap between AUROCs and clinical benefit?

## Building out the implementation science of AI

Limited user adoption—due to lack of clinician trust and model interpretability among many other reasons—has long been cited as a key barrier to clinical impact^2,3^. Encouraging providers to thoughtfully incorporate a model’s prediction into their decision and ultimate behavior regarding patient care—particularly in scenarios where predictions by the model and the human diverge—is a challenge with no clear solution yet. However, significant hurdles remain even after clinician buy-in. A successful AI tool is one that triggers a tailored workflow: the tool’s prediction must be translated into the most appropriate human intervention to generate clinical value^4^. Recent examples of clinically-impactful predictive models are ones that have been coupled with the optimal real-world intervention for each possible model output^5^. Unfortunately, little work exists on this issue: interventions are often selected somewhat arbitrarily or left up to clinician judgement^4^. We must develop methods for systematically identifying the best possible intervention to pair with an accurate prediction.

## Using real-world evidence to evaluate AI

To better understand the impact of AI at the bedside, we must embrace new ways of evaluating it. To date, there have been few randomized trials on this topic, as highlighted by Zhou et al. However, traditional time-consuming and costly RCTs are not the only way to measure the impact of these tools. To hasten the pace and lower the costs of answering this question, we must also leverage rich sources of observational data (e.g. administrative claims databases and electronic health records [EHRs]) and causal inference methods to passively monitor the impact of AI in clinical practice, as an adjunct to clinical trials. The US Food and Drug Administration (FDA) has begun using real-world data to inform regulatory decisions for drugs and devices^6^; researchers studying AI should similarly adopt this approach.

## Exploring new applications of AI

Zhou et al. reveal that the scope of applications of AI at the bedside has been almost entirely limited to making individual diagnostic and prognostic predictions; the primary outcomes for trials evaluating AI have been limited to performance on specific clinical tasks (e.g., adenoma detection rate on endoscopy); and superiority in these trials has typically been defined as exceeding human performance. To uncover additional opportunities for AI to create value for health systems, researchers must be more flexible in identifying potential use cases, selecting outcomes of interest, and defining clinical benefit. Providing targeted outreach to vulnerable patients^7^, enabling rapid comparative effectiveness studies at the bedside^8^, and automating burdensome administrative tasks^9^ should be further explored as applications for AI. Improving population health metrics^7^, reducing administrative costs^10^, and alleviating constraints on providers’ resources and time should be examined as outcomes in future trials. Furthermore, the narrow definition of a beneficial AI tool as one that outcompetes the human should be expanded to include one that effectively complements the human—either by matching human performance on repetitive tasks, or by forming a synergistic human-computer intervention that accomplishes beyond what either could do alone^11,12^.

The findings by Zhou et al. highlight several important opportunities to advance the field of clinical AI. Expanded applications, broader definitions of clinical benefit, new evaluation methods, and tailored interventions are just a few of many possible considerations that may help bridge the gap between in silico predictive performance and real-world utility.

### Reporting summary

Further information on research design is available in the Nature Research Reporting Summary linked to this article.

## Supplementary information

Reporting Summary
